# Clonal dissemination of carbapenem-resistant *Klebsiella pneumoniae* ST16 co-producing NDM-1 and OXA-232 in Thailand

**DOI:** 10.1093/jacamr/dlac084

**Published:** 2022-08-16

**Authors:** Ryuichiro Abe, Yukihiro Akeda, Dan Takeuchi, Noriko Sakamoto, Yo Sugawara, Norihisa Yamamoto, Anusak Kerdsin, Yuki Matsumoto, Daisuke Motooka, Warawut Leolerd, Pitak Santanirand, Masato Suzuki, Keigo Shibayama, Kazunori Tomono, Tetsuya Iida, Shigeyuki Hamada

**Affiliations:** Japan-Thailand Research Collaboration Center on Emerging and Re-emerging Infections, Research Institute for Microbial Diseases, Osaka University, Osaka, Japan; Department of Bacterial Infections, Research Institute for Microbial Diseases, Osaka University, Osaka, Japan; Japan-Thailand Research Collaboration Center on Emerging and Re-emerging Infections, Research Institute for Microbial Diseases, Osaka University, Osaka, Japan; Department of Infection Control and Prevention, Graduate School of Medicine, Osaka University, Osaka, Japan; Department of Bacteriology I, National Institute of Infectious Diseases, Tokyo, Japan; Japan-Thailand Research Collaboration Center on Emerging and Re-emerging Infections, Research Institute for Microbial Diseases, Osaka University, Osaka, Japan; Japan-Thailand Research Collaboration Center on Emerging and Re-emerging Infections, Research Institute for Microbial Diseases, Osaka University, Osaka, Japan; Antimicrobial Resistance Research Center, National Institute of Infectious Diseases, Tokyo, Japan; Japan-Thailand Research Collaboration Center on Emerging and Re-emerging Infections, Research Institute for Microbial Diseases, Osaka University, Osaka, Japan; Antimicrobial Resistance Research Center, National Institute of Infectious Diseases, Tokyo, Japan; Department of Infection Control and Prevention, Graduate School of Medicine, Osaka University, Osaka, Japan; Faculty of Public Health, Kasetsart University, Chalermphrakiat Sakon Nakhon Province Campus, Sakon Nakhon, Thailand; Department of Infection Metagenomics, Research Institute for Microbial Diseases, Osaka University, Osaka, Japan; Department of Infection Metagenomics, Research Institute for Microbial Diseases, Osaka University, Osaka, Japan; Faculty of Medicine Ramathibodi Hospital, Mahidol University, Bangkok, Thailand; Faculty of Medicine Ramathibodi Hospital, Mahidol University, Bangkok, Thailand; Antimicrobial Resistance Research Center, National Institute of Infectious Diseases, Tokyo, Japan; Department of Bacteriology II, National Institute of Infectious Diseases, Tokyo, Japan; Department of Infection Control and Prevention, Graduate School of Medicine, Osaka University, Osaka, Japan; Japan-Thailand Research Collaboration Center on Emerging and Re-emerging Infections, Research Institute for Microbial Diseases, Osaka University, Osaka, Japan; Department of Bacterial Infections, Research Institute for Microbial Diseases, Osaka University, Osaka, Japan; Faculty of Medicine Ramathibodi Hospital, Mahidol University, Bangkok, Thailand; Center for Infectious Disease Education and Research, Osaka University, Osaka, Japan; Japan-Thailand Research Collaboration Center on Emerging and Re-emerging Infections, Research Institute for Microbial Diseases, Osaka University, Osaka, Japan

## Abstract

**Background:**

*Klebsiella pneumoniae* ST258 and ST11 carrying *bla*_KPC_ are among the most widespread carbapenem-resistant *K. pneumoniae* strains worldwide. Our carbapenem-resistant Enterobacteriaceae surveillance in Thailand revealed a nationwide dissemination of *K. pneumoniae* ST16 isolates carrying *bla*_NDM-1_ and *bla*_OXA-232_.

**Objectives:**

To analyse the genomic details of this nationwide dissemination by focusing on plasmids and virulence factors.

**Methods:**

Using WGS data of 119 *K. pneumoniae* ST16 isolates carrying *bla*_NDM-1_ obtained in our previous surveillance study, clonality of chromosomes and plasmids of the isolates with carriage of virulence factors was evaluated.

**Results:**

Of the 119 isolates, 111 carried plasmid pKP151_NDM1, and all 104 isolates harbouring *bla*_OXA-232_ carried plasmid pKP151_OXA232. These 104 K. pneumoniae ST16 isolates showing chromosomal clonality possessed both pKP151_NDM1 and pKP151_OXA232, demonstrating clonal dissemination of K. pneumoniae ST16 with these plasmids. The isolates had essentially similar virulence factors as those of K. pneumoniae ST16 clones carrying *bla*_KPC_, which were recently reported as highly invasive clones in Brazil.

**Conclusions:**

The potential global dissemination of these invasive clones with resistance to several antibiotics highlights the importance of appropriate monitoring and strict standard precautions.

## Introduction

Among MDR Enterobacteriaceae isolates, which are being disseminated worldwide, carbapenem-resistant *Klebsiella pneumoniae* (CRKP) is of major concern because alternative treatment options, even for common bacterial infections, are limited. Carbapenem resistance is primarily conferred by carbapenemases such as KPC, OXA-48 and NDM, which hydrolyse carbapenems. *K. pneumoniae* of the clonal group (CG) 258, including ST258 and ST11, carrying *bla*_KPC_, is the most frequently reported CRKP worldwide.^1^ Outbreaks of *K. pneumoniae* ST11 and ST258 carrying *bla*_NDM-1_^2^ and *bla*_NDM-5_,^3^ respectively, have recently been sporadically reported, implying a worldwide clonal spread of *K. pneumoniae* CG258 acquiring various carbapenemase genes. Meanwhile, Andrey *et al*.^4^ reported an outbreak of *K. pneumoniae* ST16 producing KPC-2, associated with a higher mortality rate than *K. pneumoniae* ST258 clones. Global dissemination of *K. pneumoniae* ST16 carrying both *bla*_NDM-1_ and *bla*_OXA-232_ has been reported,^5,6^ calling for careful monitoring of these highly virulent clones. During our nationwide surveillance of carbapenem-resistant Enterobacteriaceae (CRE) isolates in Thailand, we identified 119 *K. pneumoniae* ST16 isolates carrying *bla*_NDM-1_.^7^ Here, we analysed the clonality of chromosomes and plasmids of these isolates and evaluated virulence factors shared among them.

## Materials and methods

### Bacterial isolates and antimicrobial susceptibility testing

Previously, we conducted nationwide surveillance of CRE isolates in Thailand, covering 11 representative hospitals in six provinces, between 2012 and 2017, and collected 747 CRE isolates from various clinical specimens of 736 patients (Figure S1, available as Supplementary data at *JAC-AMR* Online).^7^ Among 493 CRE isolates carrying *bla*_NDM_ from 489 patients (confirmed using PCR and sequencing), 119 *K. pneumoniae* ST16 isolates carrying *bla*_NDM-1_ obtained from 119 patients were analysed in this study. Antimicrobial drug susceptibility was determined as previously reported.^7^

### WGS analysis

WGS of all isolates was conducted using the Illumina HiSeq 3000 (Illumina, San Diego, CA, USA) or PacBio RS II (Pacific Biosciences, Menlo Park, CA, USA) platform.^8^ Using a combination of GridION (Oxford Nanopore Technologies, Oxford, UK) and Illumina HiSeq 3000 on the representative isolate KP151 carrying both *bla*_NDM-1_ and *bla*_OXA-232_, the complete sequence of plasmids pKP151_NDM1 and pKP151_OXA232 was determined, and plasmid clonalities were investigated by mapping the raw HiSeq 3000 and PacBio RS II reads of all isolates to the sequences of pKP151_NDM1 and pKP151_OXA232, using the Burrows–Wheeler aligner.^9^ Coverages of the reference plasmids by sequence reads were calculated using SAMtools,^10^ with 90% identity and coverage cut-offs. Following the identification of antimicrobial resistance genes and annotation using ResFinder 2.1^11^ and RASTtk,^12^ the genomic structures of the plasmids were compared with those of plasmids identified with BLAST using Easyfig.^13^ Following sequence alignments using CLC Genomics Workbench 11.0.1 (CLC Bio, Aarhus, Denmark) with default settings, SNP distance analysis was performed with CSI Phylogeny 1.4,^14^ using *K. pneumoniae* strain KPNIH1 (NZ_CP008827.1) as a reference. iTOL (https://itol.embl.de/) was used to illustrate the phylogenetic trees. The virulence factors were analysed as previously reported,^4^ using 95% identity and coverage cut-offs. The virulence factors analysed are shown in Table S1.

## Results and discussion

**Figure 1.**
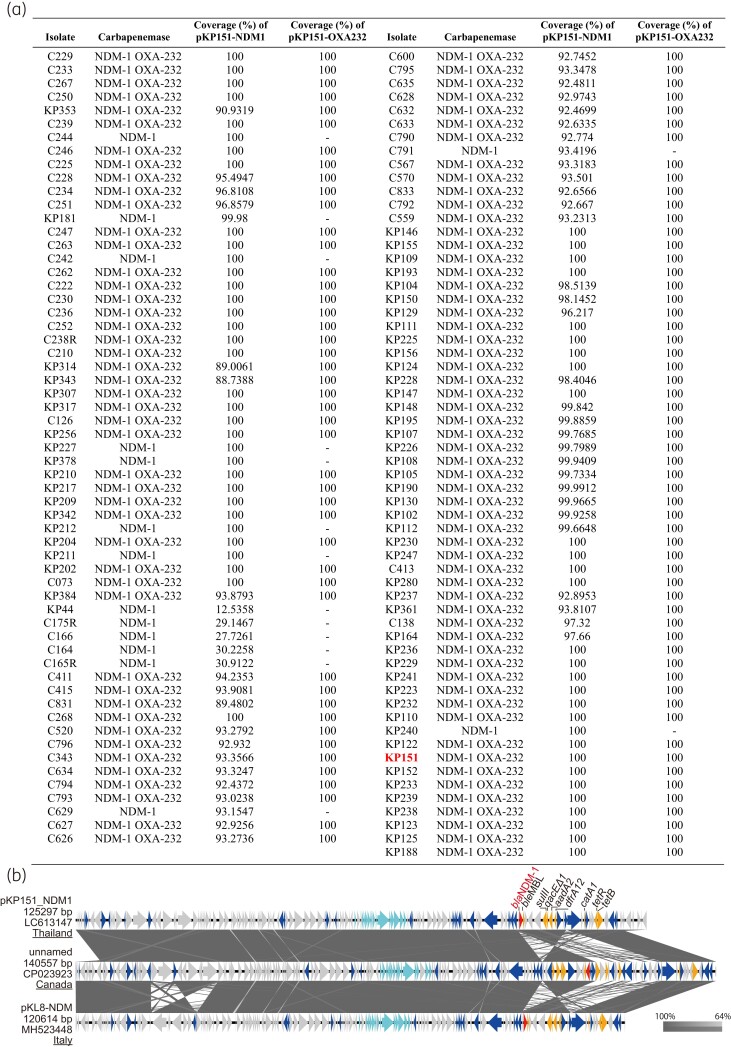
Similarity of the plasmids of study isolates and comparison with previously reported plasmids from other countries. (a) Similarity of the plasmids compared with the plasmids of KP151. Following mapping raw sequence reads obtained from HiSeq 3000 or PacBio RS II to the sequences of plasmids pKP151_NDM1 and pKP151_OXA232, coverages of the reference plasmids were calculated. (b) The structure of pKP151_NDM1 compared with that of previously reported plasmids. Block arrows indicate confirmed or putative ORFs and their orientations. Arrow size is proportional to the predicted ORF length. The colour code is as follows: red, carbapenem resistance gene; yellow, other antimicrobial resistance gene; light blue, conjugative transfer gene; blue, mobile element. Putative, hypothetical, or unknown genes are represented as grey arrows. The grey-shaded area indicates regions with high identity between sequences. Accession numbers of the plasmids are indicated below the plasmid size.

We performed comprehensive genomic analysis of 119 *K. pneumoniae* ST16 isolates carrying *bla*_NDM-1_ obtained from hospitals in five provinces in Thailand (Figure S2). First, plasmids carrying *bla*_NDM-1_ or *bla*_OXA-232_ harboured by the representative isolate KP151 were determined. The IncColKP3 plasmids carrying *bla*_OXA-232_ identified in the United States and the Netherlands were identical to pKP151_OXA232 (Figure S3), whereas the IncF1A/F1B plasmids carrying *bla*_NDM-1_, which have high similarity to pKP151_NDM1, were identified in Italy and Canada (Figure 1). This implies that both plasmids (pKP151_NDM1 and pKP151_OXA232) are disseminated widely, potentially globally. Notably, pKL8-NDM was identified from a *K. pneumoniae* ST16 isolate carrying *bla*_NDM-1_ and *bla*_OXA-232_ isolated in Italy.^5^ Plasmid clonalities of the study isolates were investigated by mapping sequence reads to the reference plasmids pKP151_NDM1 and pKP151_OXA232 (Figure 1). Of the 119 isolates, 111 carried pKP151_NDM1, and all 104 isolates harbouring *bla*_OXA-232_ carried pKP151_OXA232. SNP analysis of the core genomes revealed the chromosomal clonality of *K. pneumoniae* ST16 isolates carrying *bla*_NDM-1_, with most differences being <60 SNPs (maximum 87 SNPs, except in eight isolates), whereas six of the eight isolates that were distinct from the predominant clones did not carry *bla*_OXA-232_ (Figure S4). The majority of *K. pneumoniae* ST16 isolates showing chromosomal clonality possessed both pKP151_NDM1 and pKP151_OXA232, demonstrating clonal dissemination of *K. pneumoniae* ST16 with these plasmids.

**Figure 2.**
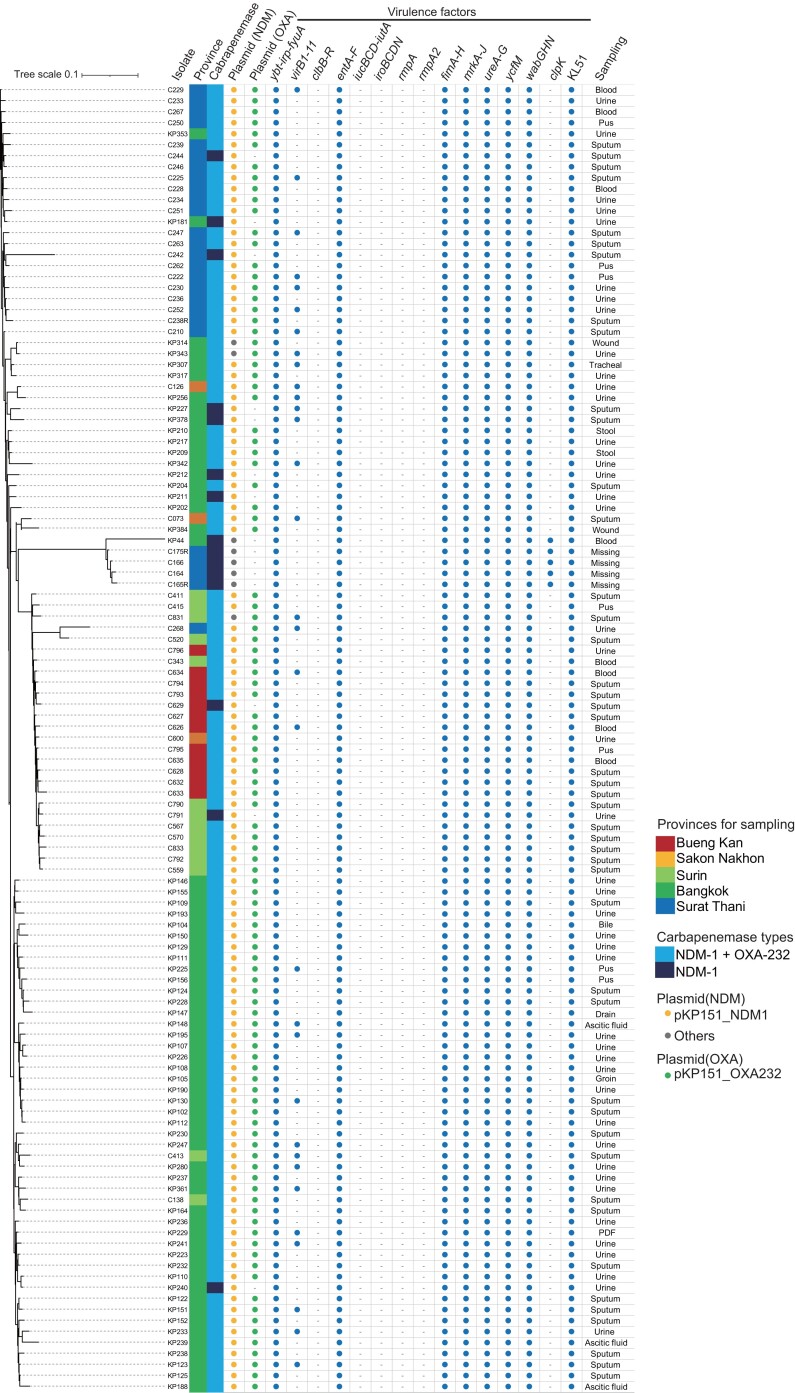
Chromosomal phylogeny, plasmidome and virulence factors of *K. pneumoniae* ST16 isolates carrying *bla*_NDM-1_. Chromosomal phylogeny was constructed using CSI Phylogeny. Samples are colour-coded according to their collection provinces (as shown in the figure). pKP151_NDM1 and pKP151_OXA232 were used as reference plasmids for sequence mapping; plasmids carrying *bla*_NDM_ distinct from pKP151_NDM1 were classified as ‘others’. Carriage of virulence genes was confirmed by aligning the contigs of sequenced genomes of each isolate to our virulence factor data set (Table S1).

The study isolates were obtained from various patient specimens, including urinary, lung, abdominal, abscess and venous blood specimens, implying that there is no infection-site specificity (Figure 2). All isolates were highly resistant to various antibiotics, including aztreonam and levofloxacin (Figure S5). Colistin and aminoglycosides are potential candidates for combination therapy, with colistin, gentamicin and amikacin susceptibility of 76.4% (91/119), 99.1% (118/119) and 100% (119/119), respectively. Furthermore, we analysed the virulence factors of these highly invasive clones (Figure 2). These isolates shared various virulence-determinant genes (*ybt-irp-fyuA*, *entA-F, fimA-H*, *mrkA-J*, *urea-G*, *wabGHN* and capsular genes of KL51) corresponding to highly invasive *K. pneumoniae* ST16 clones producing KPC, which was recently reported in Brazil.^4^ Hypervirulence genes (*iucBCD-iutA*, *iroBCDN*, *rmpA* and *rmpA2*) were not identified in the ST16 isolates in the current and previous studies, implying the involvement of unknown key factors in the ST16 virulence profile.

We report nationwide clonal dissemination of *K. pneumoniae* ST16 isolates carrying *bla*_NDM-1_ and *bla*_OXA-232_ in Thailand. These clones possess the same virulence genes as highly invasive *K. pneumoniae* ST16 clones, causing various infections. Appropriate monitoring of the global dissemination of this invasive clone with broad spectrum antimicrobial resistance and prevention of its dissemination are urgently required.

## Supplementary Material

dlac084_Supplementary_Data

## Data Availability

WGS data are available from the DNA Data Bank of Japan under the accession numbers listed in Table S2.

## Supplementary data

Figures S1 to S5 and Tables S1 and S2 are available as Supplementary data at *JAC-AMR* Online.
