# Supplementary material for: Clonal dissemination of carbapenem-resistant Klebsiella pneumoniae ST16 co-producing NDM-1 and OXA-232 in Thailand
Source: JAC Antimicrob Resist. 2022 Aug 16;4(4):dlac084. doi: 10.1093/jacamr/dlac084 (PMC9380991; doi:10.1093/jacamr/dlac084)
Supplement: dlac084_Supplementary_Data [file dlac084_supplementary_data.pdf]

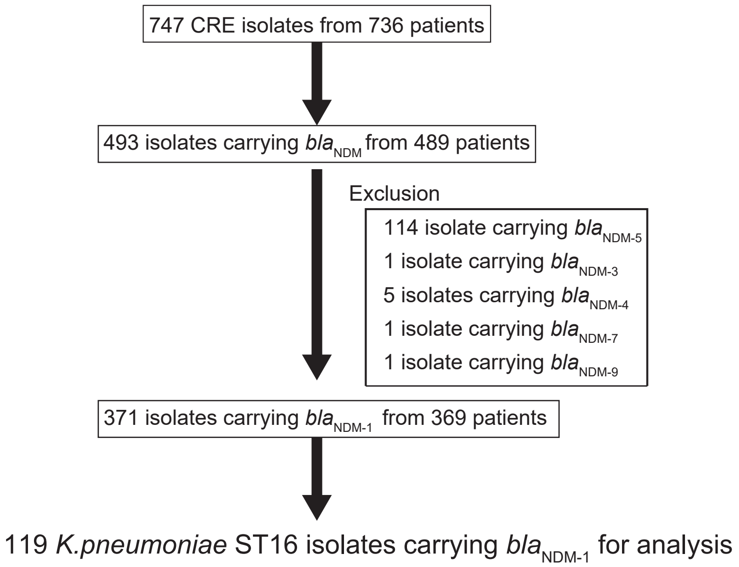

Figure S1. Consort flow diagram of the isolation of *K. pneumoniae* ST16 carrying *bla*$_{NDM-1}$.

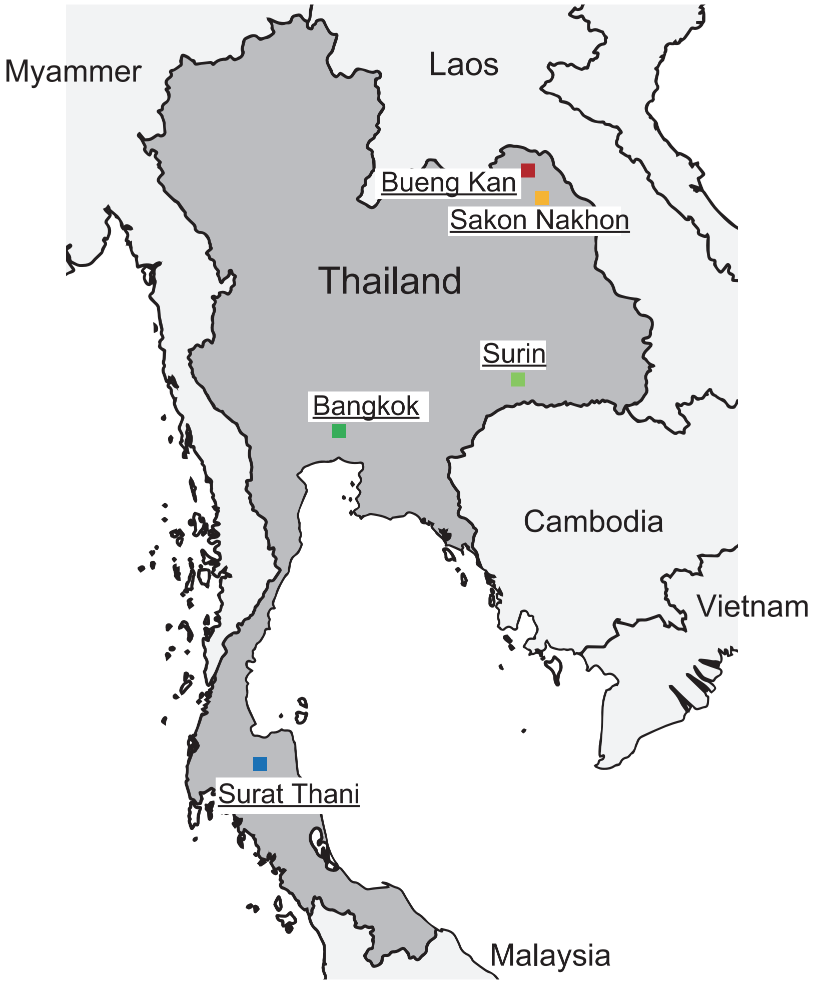

Figure S2. Locations of the provinces where the samples were collected in Thailand.

The colour codes used for the provinces correspond to those used in Figure 2.

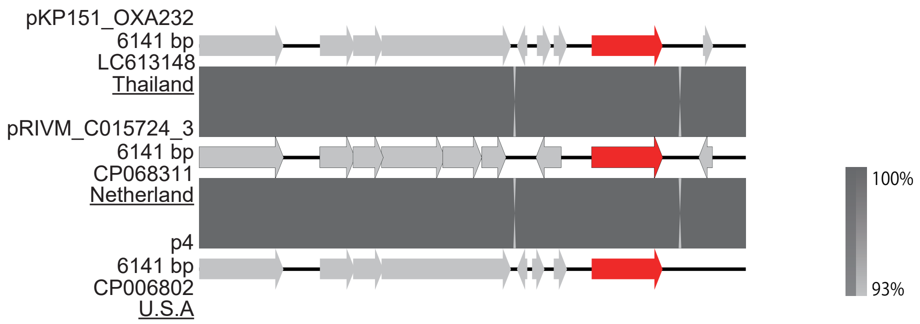

Figure S3. Comparison of plasmid structures with previously reported plasmids from other countries. pKP151_OXA232 was compared with previously reported plasmids picked up by BLAST. Block arrows indicate confirmed or putative open reading frames (ORFs) and their orientations. Arrow size is proportional to the predicted ORF length. The color code is as follows: red, carbapenem resistance gene; yellow, other antimicrobial resistance gene; light blue, conjugative transfer gene; blue, mobile element. Putative, hypothetical, or unknown genes are represented as grey arrows. The grey-shaded area indicates regions with high identity between the two sequences. Accession numbers of the plasmids are indicated below the plasmid size.

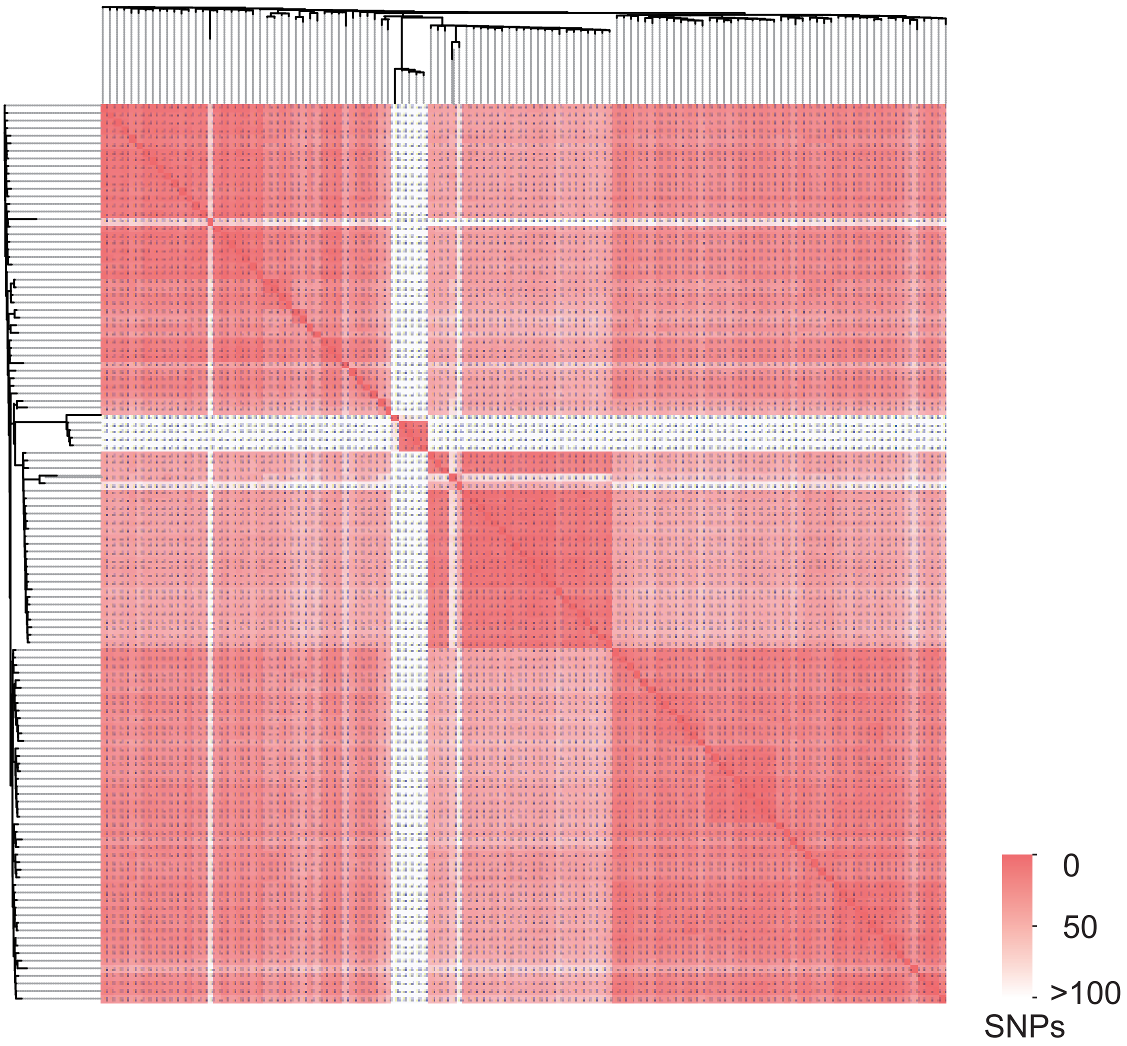

Figure S4. SNP distances among the study isolates. Heatmap of the SNP distances calculated using CSI Phylogeny, showing the clonality of *K. pneumoniae* ST16 isolates carrying bla_{NDM-1} or $bla_{OXA-232}$. Chromosomal phylogeny corresponds to Figure 1.

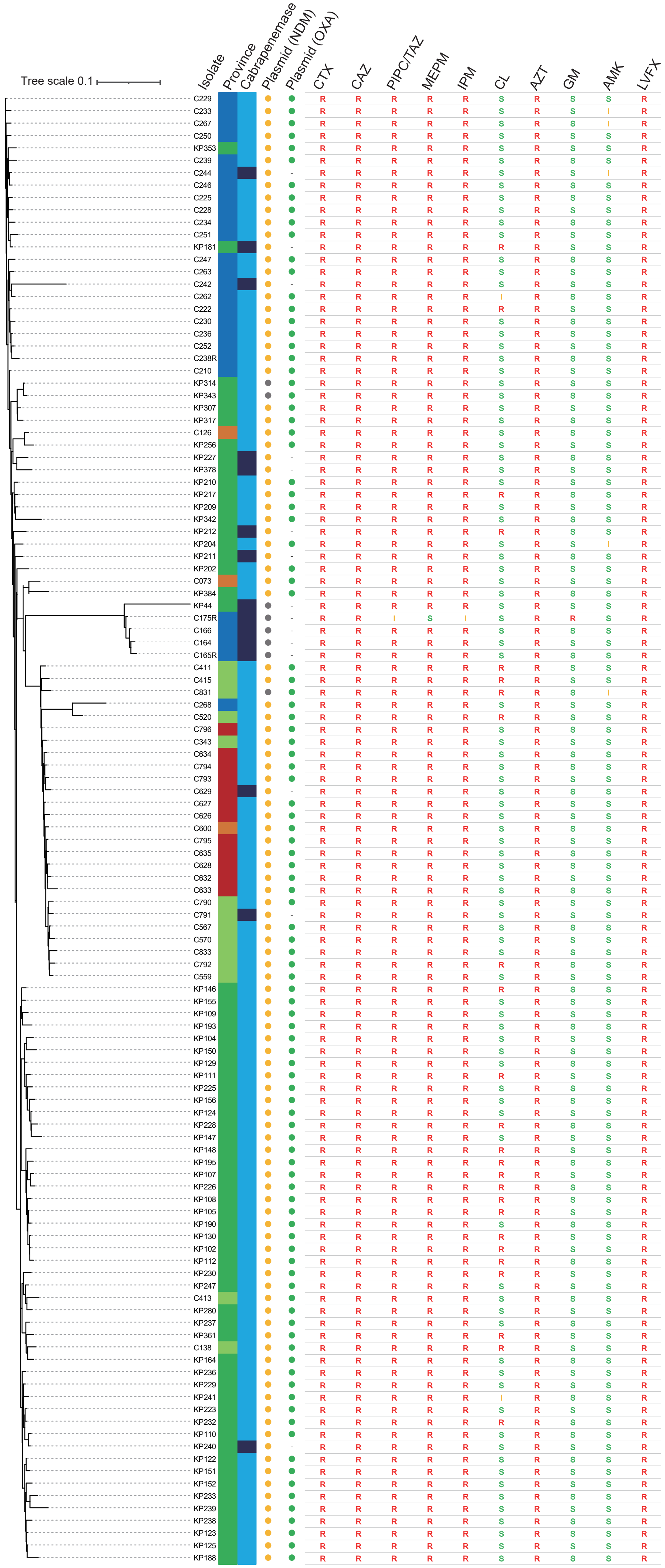

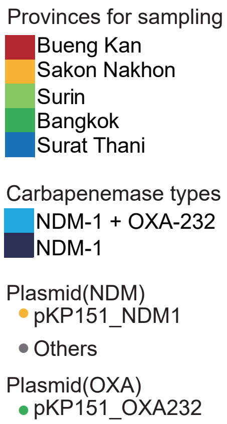

Figure S5. Antimicrobial drug susceptibility of the study isolates. Chromosomal phylogeny, sampling location (province), carbapenemases produced by the study isolates, and plasmidome analysis correspond to Figure 1. Antimicrobial resistance is indicated as follows: R, resistant; I, intermediate; S, susceptible. Abbreviations: CTX, cefotaxime; CAZ, ceftazidime; PIPC/TAZ, piperacillin/tazobactam; MEPM, meropenem; IPM, imipenem; CL, colistin; AZT, aztreonam; GM, gentamicin; AMK, amikacin; LVFX, levofloxacin.

Table S1. Virulence factors dataset used in this study.

Virulence Factor	virulence gene	Reference strain or plasmid	Accession number
Yersiniabactin	*ybtAEPQSTUX-irp1-irp2-fyuA*	*Klebsiella pneumoniae* subsp. *pneumoniae* NTUH-K2044	AP006725
Type-IV Secretion System	*virB1-11*	*Klebsiella pneumoniae* subsp. *pneumoniae* NTUH-K2044	AP006725
Colibactin	*clbBCDEFGHHIJKLMNOPQR*	*Escherichia coli strain* IHE3034	AM229678
Enterobactin	*entABCDEF*	*Klebsiella pneumoniae* subsp. *pneumoniae* NTUH-K2044	AP006725
Aerobactin	*iucBCD-iutA*	*Klebsiella pneumoniae* CG43, plasmid pLVPK	NC_005249
Salmochellin	*iroBCDN*	*Klebsiella pneumoniae* CG43, plasmid pLVPK	NC_005249
CPS transcriptional activator	*rmpA*	*Klebsiella pneumoniae* CG43	NC_005249
CPS transcriptional activator 2	*rmpA2*	*Klebsiella pneumoniae* CG43	NC_005249
Fimbria type I	*fimABCDEFGH*	*Klebsiella pneumoniae* subsp. *pneumoniae* T69	HG518478
Fimbria type III	*mrkABCDFHIJ*	*Klebsiella pneumoniae* subsp. *pneumoniae* T69	HG518478
Urease	*ureABCDEFG*	*Klebsiella pneumoniae* subsp. *pneumoniae* NTUH-K2044	AP006725
Outer membrane lipoprotein	*ycfM*	*Klebsiella pneumoniae* subsp. *pneumoniae* T69	HG518478
LPS synthesis	*wabGHN*	*Klebsiella pneumoniae* subsp. *pneumoniae* NTUH-K2044	AP006725
Thermotolerance (chaperone)	*clpK*	*Klebsiella pneumoniae* strain C132-98 plasmid	FJ042668

Table S2. Sequencing accession numbers and dates of sampling from patients.

Isolate	Sequencer	Accession Number	Sampling date
C073	HiSeq	DRX115359	2016/2/15
C126	HiSeq	DRX115404	2016/4/24
C138	HiSeq	DRX115414	2016/7/3
C164	HiSeq	DRX141053	2015/6/1
C165R	HiSeq	DRX141055	2015/6/18
C166	HiSeq	DRX141056	2015/6/2
C175R	HiSeq	DRX141065	2015/8/5
C210	HiSeq	DRX141096	2016/3/7
C222	HiSeq	DRX141102	2016/5/1
C225	HiSeq	DRX141105	2016/6/30
C228	HiSeq	DRX141107	2016/7/8
C229	HiSeq	DRX141108	2016/7/17
C230	HiSeq	DRX141109	2016/7/1
C233	HiSeq	DRX141112	2016/7/19
C234	HiSeq	DRX141113	2016/6/17
C236	HiSeq	DRX141115	2016/6/27
C238R	HiSeq	DRX141118	2016/7/2
C239	HiSeq	DRX141119	2016/7/2
C242	HiSeq	DRX141122	2016/7/9
C244	HiSeq	DRX141124	2016/7/13
C246	HiSeq	DRX141126	2016/7/27
C247	HiSeq	DRX141127	2016/6/29
C250	HiSeq	DRX141129	2016/7/16
C251	HiSeq	DRX141130	2016/8/1
C252	HiSeq	DRX141131	2016/8/1
C262	HiSeq	DRX141140	2016/8/23
C263	HiSeq	DRX141141	2016/8/12
C267	HiSeq	DRX141145	2016/8/29
C268	HiSeq	DRX141146	2016/8/29
C343	HiSeq	DRX141209	2016/11/1
C411	HiSeq	DRX141276	2016/11/24
C413	HiSeq	DRX141278	2016/11/24
C415	HiSeq	DRX141280	2016/11/25
C520	HiSeq	DRX141335	2017/2/18
C559	HiSeq	DRX141363	2017/3/24
C567	HiSeq	DRX141366	2017/4/4
C570	HiSeq	DRX141368	2017/3/28
C600	HiSeq	DRX141397	2017/3/29
C626	HiSeq	DRX141400	2017/4/10
C627	HiSeq	DRX141401	2017/4/17
C628	HiSeq	DRX141402	2017/4/20
C629	HiSeq	DRX141403	2017/4/22
C632	HiSeq	DRX141406	2017/5/1
C633	HiSeq	DRX141407	2017/5/5
C634	HiSeq	DRX141408	2017/4/25
C635	HiSeq	DRX141409	2017/4/29
C790	HiSeq	DRX141485	2017/6/14
C791	HiSeq	DRX141486	2017/6/15
C792	HiSeq	DRX141487	2017/6/15
C793	HiSeq	DRX141488	2017/5/10
C794	HiSeq	DRX141489	2017/5/17
C795	HiSeq	DRX141490	2017/5/23
C796	HiSeq	DRX141491	2017/5/18
C831	HiSeq	DRX141514	2017/7/11
C833	HiSeq	DRX141516	2017/7/6
KP102	HiSeq	DRX115079	2015/12/20
KP104	HiSeq	DRX115081	2015/12/21
KP105	HiSeq	DRX115082	2015/12/21
KP107	HiSeq	DRX115084	2015/12/25
KP108	HiSeq	DRX115085	2015/12/21
KP109	HiSeq	DRX115086	2015/12/21
KP110	HiSeq	DRX115087	2015/12/25
KP111	HiSeq	DRX115088	2015/12/25
KP112	HiSeq	DRX115089	2015/12/23
KP122	HiSeq	DRX115091	2015/12/30
KP123	HiSeq	DRX115092	2016/1/1
KP124	HiSeq	DRX115093	2016/1/7
KP125	HiSeq	DRX115094	2016/1/12
KP129	HiSeq	DRX115098	2016/1/22
KP130	HiSeq	DRX115099	2016/1/14
KP146	HiSeq	DRX115104	2016/8/2
KP147	HiSeq	DRX115105	2016/4/2
KP148	HiSeq	DRX115106	2016/1/2
KP150	HiSeq	DRX115108	2016/1/2
KP151	HiSeq	DRX115109	2016/2/2
KP152	HiSeq	DRX115110	2016/2/2
KP155	HiSeq	DRX115113	2016/1/31
KP156	HiSeq	DRX115114	2016/1/31
KP164	HiSeq	DRX115118	2016/7/3
KP181	HiSeq	DRX115120	2016/3/15
KP188	HiSeq	DRX115121	2016/3/17
KP190	HiSeq	DRX115123	2016/3/14
KP193	HiSeq	DRX115126	2016/2/19
KP195	HiSeq	DRX115128	2016/3/19
KP202	HiSeq	DRX114777	2016/1/4
KP204	HiSeq	DRX115130	2016/4/4
KP209	HiSeq	DRX115132	2016/4/25
KP210	HiSeq	DRX115133	2016/4/25
KP211	HiSeq	DRX115134	2016/4/25
KP212	HiSeq	DRX115135	2016/4/25
KP217	HiSeq	DRX115136	2016/4/19
KP223	HiSeq	DRX115138	2016/3/25
KP225	HiSeq	DRX115140	2016/4/2
KP226	HiSeq	DRX115141	2016/4/4
KP227	HiSeq	DRX115142	2016/6/4
KP228	HiSeq	DRX115143	2016/5/4
KP229	HiSeq	DRX115144	2016/8/4
KP230	HiSeq	DRX115145	2016/12/4
KP232	HiSeq	DRX115147	2016/4/18
KP233	HiSeq	DRX115148	2016/12/5
KP236	HiSeq	DRX115149	2016/4/26
KP237	HiSeq	DRX115150	2016/4/25
KP238	HiSeq	DRX115151	2016/2/5
KP239	HiSeq	DRX115152	2016/4/5
KP240	HiSeq	DRX115153	2016/6/5
KP241	HiSeq	DRX115154	2016/7/5
KP247	HiSeq	DRX114785	2016/5/13
KP256	HiSeq	DRX114786	2016/1/5
KP280	HiSeq	DRX141737	2016/8/10
KP307	HiSeq	DRX141743	2016/9/18
KP314	HiSeq	DRX141746	2016/10/23
KP317	HiSeq	DRX141748	2016/10/20
KP342	HiSeq	DRX141755	2016/11/3
KP343	HiSeq	DRX141756	2016/10/29
KP353	HiSeq	DRX141760	2016/10/11
KP361	HiSeq	DRX141762	2016/11/18
KP378	HiSeq	DRX141772	2017/1/10
KP384	HiSeq	DRX141774	2017/1/9
KP44	PacBio	DRX114738	2014/6/10
